# Efficient introgression of allelic variants by embryo-mediated editing of the bovine genome

**DOI:** 10.1038/srep11735

**Published:** 2015-07-09

**Authors:** Jingwei Wei, Stefan Wagner, Dan Lu, Paul Maclean, Daniel F. Carlson, Scott C. Fahrenkrug, Götz Laible

**Affiliations:** 1 AgResearch, Ruakura, Hamilton, New Zealand; 2 Guangxi University, Nabbing, China; 3 China Agricultural University, Beijing, China; 4 Recombinetics, St. Paul, Minnesota, USA

## Abstract

The recent development of designer nucleases allows for the efficient and precise introduction of genetic change into livestock genomes. Most studies so far have focused on the introduction of random mutations in cultured cells and the use of nuclear transfer to generate animals with edited genotypes. To circumvent the intrinsic uncertainties of random mutations and the inefficiencies of nuclear transfer we directed our efforts to the introduction of specific genetic changes by homology-driven repair directly in *in vitro* produced embryos. Initially, we injected zinc finger nuclease (ZFN)-encoding mRNA or DNA into bovine zygotes to verify cleavage activity at their target site within the gene for beta-lactoglobulin (*LGB*) and detected ZFN-induced random mutations in 30% to 80% of embryos. Next, to precisely change the *LGB* sequence, we co-injected ZFNs or transcription activator-like effector nucleases (TALENs) with DNA oligonucleotides (ODNs). Analysis of co-injected embryos showed targeted changes in up to 33% (ZFNs) and 46% (TALENs) of blastocysts. Deep sequence analysis of selected embryos revealed contributions of the targeted *LGB* allele can reach 100% which implies that genome editing by zygote injections can facilitate the one-step generation of non-mosaic livestock animals with pre-designed biallelic modifications.

The emergence of designer nucleases such as ZFNs, TALENs and most recently clustered, regularly interspaced, short palindromic repeat (CRISPR)/CRISPR-associated (Cas) nucleases has greatly improved our abilities for the controlled introduction of genetic modifications. Harnessing the cellular repair mechanisms of non-homologous end joining (NHEJ) and homology directed repair in response to damages to the integrity of the genome caused by double strand breaks, designer nucleases have been used for the generation of gene knockouts, targeted deletions or insertions and replacements ranging from small nucleotide polymorphisms (SNPs) to the exchange of entire genes (Fig. 1, reviewed in^1^). The application of genome editing for the generation of genetically-modified animals provides the option of two alternative approaches: transfection of cells combined with somatic cell nuclear transfer (SCNT); and injection of embryos, followed by their transfer to surrogate recipients. Zygote injections are the method of choice in rodents or in rabbits despite the limited control of this approach over the extent of the introduced sequence changes. In these species, failure to generate biallelically-modified embryos (to produce functional knockout animals, for example) in a single step does not constitute a substantial hurdle. The intermediate generation of heterozygous or mosaic animals can be quickly remedied by breeding from animals that transmit the modification to the next generation. In contrast, in livestock species with considerably longer gestation periods and a typically lower number of offspring (apart from pigs and chickens), SCNT has so far been the preferred route. As the main advantage, this approach allows for the full characterization of the edited genomes, including the detection of any potential off target events, and identification of suitable biallelically modified donor cells that can be used to generate the desired animals^2^,^3^,^4^,^5^. SCNT, however, is often hampered by a high incidence of developmental abnormalities that are associated with substantial welfare concerns and results in low production efficiencies of cloned offspring, counteracting the aforementioned advantages compared with zygote injections. Recently, Lillico *et al.*^6^ showed that the zygote injection pathway can be successfully applied in livestock for the introduction of random insertions or deletions (indels) mediated by the error NHEJ repair of DNA double strand breaks. TALEN and ZFN injections in porcine zygotes resulted in both mono- and biallelic modifications of the *RelA* gene and biallelically-modified piglets were born^6^. The targeted gene disruption by embryo injection was subsequently further extended to sheep and cattle^7^ and was also demonstrated for the CRISP/Cas9 system in pigs^8^,^9^.

In this study, we aimed to develop a more efficient genome editing approach that uses i) injection of ZFNs and TALENs for producing embryos, unburdened by the reprogramming-related inefficiencies associated with SCNT and ii) introduction of precise template-encoded mutations, avoiding the wasteful generation of large numbers of undesired, random mutations potentially not including modifications required for the intended functional outcome. We used the bovine *LGB* gene, encoding the allergenic milk protein beta-lactoglobulin (BLG), as our model target locus which was previously shown to be amenable to ZFN-mediated modification in cultured bovine somatic cells^10^. We show that ZFN and TALENs injections in bovine zygotes result in a high percentage of blastocysts carrying random mutations that disrupt the *LGB* locus. Extending on this, we demonstrate for the first time that ZFN and TALENs co-injections with homology repair templates result in highly efficient introgression of precise ODN-mediated mutations in a livestock species.

## Results

### Embryo-mediated introduction of random indels

**Figure 2:**
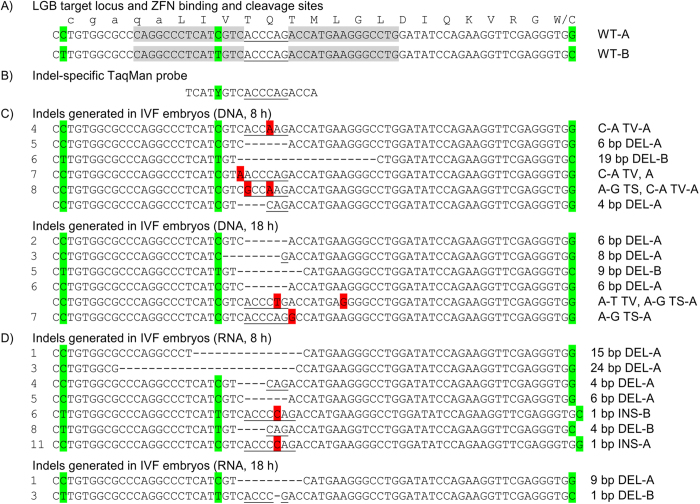
*LGB* target locus and target-specific indels generated by ZFN injection into one cell embryos. (**A**) Shown is the DNA sequence for the relevant region of the *LGB* target locus for the two main wild type *LGB* variants A (WT-A) and B (WT-B). The encoded amino acids of BLG are given in single letter code above the DNA sequence with lower case indicating amino acids of the signal peptide and upper case amino acids of the mature protein. Nucleotides highlighted in green depict polymorphic sites differing in WT-A and WT-B. The ZFN binding sites are indicated by grey boxes and the ZFN cleavage site is underlined. (**B**) Sequence of the TaqMan probe used for the mutation specific PCR assay and its position relative to the *LGB* locus. At the polymorphic site, the probe contains a pyrimidine (either C or T) shown as Y. (**C**) Sequence of mutated alleles generated by injection of DNA-encoded ZFNs into individual IVF embryos, numbered on the left. The mutations are detailed on the right with size in bp, deletion (DEL), insertion (INS), transversions (TV) and transitions (TS) and in which *LGB* variant (A or B) the mutation was introduced. Sequence changes of DELs are indicated as dash and additional nucleotides and other point mutations are highlighted in red. D) Mutations generated by injection of RNA-encoded ZFNs into individual IVF embryos.

For our study we first used a ZFN pair which binds to a target sequence in exon 1 of the bovine *LGB* locus and cleaves within the sequence encoding amino acids four and five of the mature BLG protein (Fig. 2A). The ZFN pair was designed to target one of the two main wild type alleles, variant B which differs from variant A by three SNPs within the target region, one of which is located within the binding site for the 5’ ZFN monomer (Fig. 2A). The ZFNs had been previously activity-verified for the successful introduction of indels in both *LGB* variants in primary bovine cells^10^. To determine whether these ZFNs could also induce mutations in bovine embryos produced by *in vitro* fertilization (IVF), we co-injected plasmid DNA or *in vitro*-transcribed polyadenylated RNA encoding the ZFN pair and GFP RNA into the cytoplasm of zygotes. We chose to inject at two different time points: 8 h (immediately after completion of IVF) and 18 h (successfully used previously^11^) post fertilization, reasoning that an earlier time point for injections may result in earlier expression and avoid or limit the generation of mosaic embryos. About 11% to 12% of zygotes co-injected with ZFN DNA and GFP RNA developed to blastocysts (Table 1). Co-injections using RNA-encoded ZFNs in combination with GFP RNA showed slightly better development rates with about one third of injected zygotes developing into blastocysts. GFP expression proved to be an unreliable reporter for indirectly indicating ZFN expression in co-injected embryos. While uniform GFP expression was readily detectable in most or all blastocysts in some experiments (Fig. S1), in other co-injection experiments none of the blastocysts showed detectable GFP expression even though most of these blastocysts carried ZFN-triggered mutations (Table 1). Blastocysts developed from injected zygotes were analyzed for random, ZFN-triggered mutations at the ZFN cut site, initially with the most commonly used Cel-I (Transgenomic)^12^ or T7E1 (New England Biolabs)^13^ mismatch cleavage assays. In both assays, detection of cleavage products indicates the presence of ZFN-induced mutations at the target site. We did, however, consistently observe high background levels of mismatch cleavage bands in samples of control blastocysts developed from non-injected zygotes (data not shown), which may be due to mismatch cleavage in “*LGB* heterozygous” embryos containing wild type allele variants A and B. Because this rendered the results unreliable, we alternatively developed a TaqMan PCR assay with a TaqMan probe designed to only bind the known wild type sequence (Fig. 2B) while the occurrence of indels or SNPs in or near the ZFN cut site prevents binding of the probe. With the expectation that blastocysts derived from injected zygotes will carry variable ratios of wild type and mutated alleles, fragments of the *LGB* target locus were amplified from blastocysts and subcloned into a plasmid prior to analysis. Following transformation into bacteria, individual bacterial colonies were analyzed with the TaqMan assay and melt temperature assay of the PCR amplicons to identify the presence of genome-edited sequences (Fig. S2). Analysis of a total of thirty IVF blastocysts, co-injected at the zygote stage, revealed that a high percentage of blastocysts (including GFP-negative blastocysts), ranging from 29% to 83% for RNA and DNA injections at the two time points, carried mutations (Table 1). None of the treatment groups appeared to offer superior efficiencies with no significant differences being detectable between the different experimental conditions.

For subsequent sequence analysis, we selected subclones that were identified to contain the sequence of a mutated allele and up to ten subclones for each confirmed blastocyst were sequenced (Figs 2C,D). Across all treatment groups, we detected small (1–24 bp) deletions (67%), some point mutations, either at or close to the ZFN cleavage site (24%) and a few one bp insertions (10%). For some of the analyzed blastocysts (2/19) we detected the presence of two, differently mutated alleles.

### Embryo-mediated introduction of precise genome edits

Next, we aimed to generate precise mutations that would result in a knockout of the target gene. This strategy avoids reliance on the unpredictable nature of NHEJ-generated random mutations that often fail to disrupt the reading frame. To introduce precise mutations, we co-injected ZFNs together with a single stranded ODN as homology repair template. ODN 970 contains a 5 bp insertion which creates a new XbaI site at the ZFN cleavage site and disrupts the reading frame of the *LGB* gene after the first five amino acids of the mature protein (Fig. S3). Because the five bp insertion does increase the spacer between the two ZFN binding sites, the successfully genome-edited locus is no longer an efficient target for the ZFNs. Zygotes were co-injected 8 h or 18 h post IVF with plasmids encoding ZFNs and ODN 970. Embryo development to the blastocyst stage ranged from 10% to 19% (Table 1).

**Figure 3:**
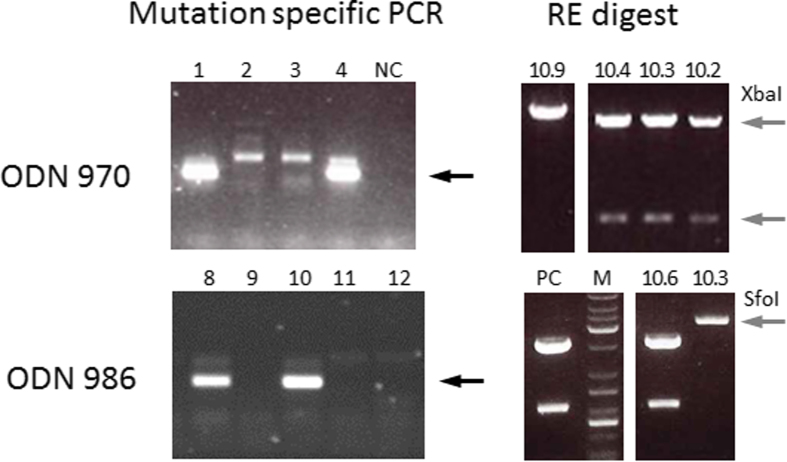
Detection of ODN-mediated genome edits by nested PCR and restriction mapping. Shown are representative examples of PCR results for the second, mutation-specific fragment amplification from individual blastocysts that were co-injected with ZFN and ODN 970 or 986 (left hand panels) at the zygote stage. Black arrows indicate successful amplification that identifies genome-edited embryos. NC: negative control (water). The right hand panels display the result of diagnostic restriction enzyme (RE) digests of different subclones (10.2, 3, 4, 9) derived from embryo 10 co-injected with ZFN/ODN 970 and subclones 10.3 and 10.6 derived from ZFN/ODN 986 injected embryo10. The grey arrows point to diagnostic restriction fragments that differ due to the presence of a new XbaI or absence of a SfoI site in the genome edited allele. PC: positive control (wild type blastocyst) M: DNA size marker.

In contrast to the detection of random indels, the presence of precise ODN-mediated mutations can be readily identified by the presence of the newly introduced XbaI restriction site or by a nested PCR strategy relying on one internal primer designed to exclusively bind the mutated *LGB* allele. First, the exon 1 target region, potentially containing a precise ODN-induced mutation, was amplified from blastocysts derived from co-injected zygotes. The ~550 bp amplicons were then screened with a mutation-specific, nested PCR to identify genome-edited blastocysts. This revealed that co-injections of ODN 970 with DNA-encoded ZFNs efficiently generated precisely genome-edited blastocysts (Fig. 3). Template-specified mutations were detected in 18% and 33% of blastocysts derived from co-injections at 8 h and 18 h, respectively (Table 1). For these particular injections, we used variants of our standard ZFNs with plasmid constructs that expressed the ZFNs as fusion proteins with red and green fluorescent protein (ZFN-FL). Repeats of co-injections with our standard ZFNs and ODN produced comparable results (data not shown). Next, the amplified fragments from blastocysts that were positively identified for containing the precise mutation were subcloned into a plasmid vector. Individual plasmids were then subjected to Xba I restriction revealing digest patterns indicative of both mutated (two fragments of 4.2 kb and 350 bp) and wild type alleles (linearized plasmid) for different subclones of a specific blastocyst (Fig. 3). The introduced genome edits were further corroborated by sequencing subclones from a selected embryo sample. This revealed that the intended sequence changes were present with some of the subclones containing additional point mutations next to the ZFN cut site (Fig. S4A).

To demonstrate that the successful introduction of a precise genome edit is not dependent on the unique sequence of a particular ODN, we next attempted to introduce a small deletion with a different ODN as template. ODN 986 specifies a 9 bp deletion that creates a new stop codon and disrupts the BLG reading frame within the BLG signal peptide (Fig. S3). While the edited sequence will lack a SfoI restriction site compared to the unmodified locus, it is less clear whether this will significantly affect the binding affinity of the 5’ ZFN monomer. Similar to the results for ODN 970, analysis of blastocysts developed from zygotes that were co-injected with ODN 986 by edit-specific PCR, SfoI restriction digest and sequencing of subclones with mutant alleles demonstrated the successful introgression of the template-defined mutation (Fig. 3, Table 1, Fig. S4B). For co-injections with DNA-encoded ZFNs, template-specified mutations were detected in 27% to 33% of injected embryos for the two injection times (Table 1). Co-injections with ODN 986 were also performed with RNA-encoded ZFNs. Here, introgression rates were between 12% and 40% for the two injection times (Table 1). Statistical analysis comparing the different treatment groups showed that the mutation rate was not significantly different between any of the tested experimental factors.

Extending on our injections with ZFN, we used two TALEN pairs that target the sequences immediately downstream of the *LGB* start codon for the introgression of the 9 bp deletion specified by ODN 986 (Fig. S5). Successful introgression of the deletion generates alleles that are no longer efficient targets for the TALEN pairs. The deletion reduces the spacer region between the 5’ and 3’ TALEN monomers of btBLG 1.1 and removes half of the binding site of the 3’ monomer. For the TALEN pair btBLG 1.2, activity is affected by a more severe reduction of the spacer length from 16 to seven nucleotides (Fig. S5). Embryo development of co-injected zygotes to the blastocyst stage was between 44% (btBLG 1.1) and 31% (btBLG 1.2) for co-injections with TALEN plasmids (Table 1). Blastocysts were analyzed with the mutation-specific PCR assay which demonstrated that both TALEN pairs facilitate the introgression of the ODN 986-specified deletion with efficiencies of 40% (btBLG 1.1) to 46% (btBLG 1.2) (Table 1). The embryo development rate for co-injection of ODN 986 with RNA-encoded btBLG 1.2 was 29% and similar to DNA co-injections (Table 1). Detection of introgression in RNA-injected embryos verified the genome editing activity of RNA-encoded btBLG 1.2, albeit with a significantly lower efficiency (11%, p < 0.001). To further substantiate the PCR evidence of the introduced 9 bp deletion, amplified fragments of the *LGB* locus from selected embryos were subcloned into plasmid vectors and verified by analytical restriction digests. For two embryos, sequencing of subcloned *LGB* fragments with confirmed edits revealed precise introgression of the ODN 986-specified mutation and the additional presence of a point mutation in embryo 2 that was derived from an RNA-injection (Fig. S4C).

### Prevalence of mutated and wild type alleles in co-injected embryos

So far our results demonstrated the successful introduction of indels and precise mutations into the genomes of injected embryos. Due to the limitations of the subclone analysis to only relatively small numbers, it remained unclear, whether all, most or only a few cells of the injected embryos were genome-edited. To determine the overall prevalence of mutated and wild type alleles within an entire embryo, we utilized deep sequencing of target locus amplicons from blastocysts derived from injected zygotes. We selected nine embryos that were injected with only ZFNs and 12 ZFN/ODN 986 and 18 TALEN (btBLG1.2)/ODN 986 co-injected embryos and analyzed the overall distribution of edited and wild type alleles in individual embryos.

Consistent with the results from the limited sequencing of subcloned fragments, deep sequencing revealed the introduction of various small deletions and insertions and single point mutations in embryos injected only with ZFNs (Table 2, Table S1A). While some embryos such as Z14 contained two edited alleles with different deletions, the presence of three polymorphic sites in the sequence of ODN 986 (see Fig. 2 and S4) can generate further allelic variants (Table S1A, Z14 and Z16), perhaps indicative of several independent editing events. The more in-depth sequence analysis demonstrated that injection of both RNA- and DNA-encoded ZFNs can result in high editing efficiencies and generated embryos (Z14, Z17 and Z22) that were comprised of only edited alleles, no longer containing a wild type allele (Table 2). However, not all embryos showed complete conversion of wild type to edited alleles in all cells. RNA-injected embryos Z15 and Z16 showed high to moderate counts for the edited alleles (83% and 54%) and corresponding levels of the wild type alleles (16% and 39%). In particular DNA-injection resulted in embryos (Z18, Z19, Z24) that contained only few cells with an edited allele limiting the sequence reads for the edited allele to small fractions of between 2% and 12%.

**Figure 4:**
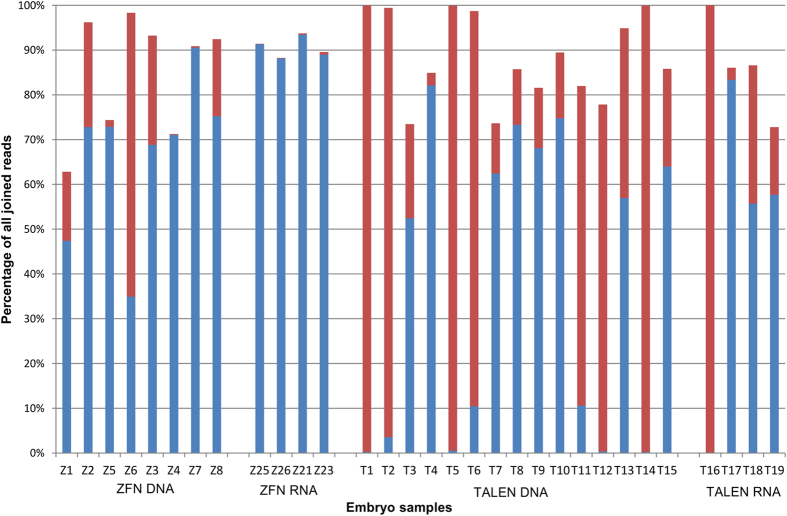
Deep sequencing analysis of ODN 986 co-injected embryos. Selected embryos derived from both ZFN/ODN 986 and TALENs/ODN 986 co-injections were analyzed for the presence of ODN-triggered genome edits and wild type sequencing reads. The representation of alleles with an ODN 986-specified 9 bp deletion (red) and combined wild type alleles A and B (blue) are depicted as percentage of all joined paired end sequence reads for individual embryos injected with DNA- or RNA-encoding ZFNs or TALENs as indicated. The underlying values used to generate the graph can be found in Table S2. Total percentages markedly below 100% denote the presence of additional sequence variants that are not included in the figure and are detailed in Table S1.

Analogous to random ZFN-induced mutations, we determined the efficiency of ODN 986-mediated introgression of the specific 9 bp deletion (Fig. 4, Table S1B, Table S2). With DNA-encoded ZFNs, the frequency of the precisely edited allele was as high as 63% in embryo Z6 indicating a high homology-directed editing potential in microinjected zygotes. However, the efficiency fluctuated greatly between different embryos with some (Z4 and Z7) showing a contribution of the edited allele of below 1%. Injections of RNA-encoded ZFNs proved less successful for introgression of the deletion, as these embryos contained notably fewer mutated alleles (frequency of 0.1 to 0.6%) than their DNA-injected counterparts (Fig. 4, Table S2).

Introgression of the 9 bp deletion worked markedly better with TALENs-injected embryos. Within the subset of 15 embryos that were co-injected with the TALEN plasmids, approximately half of the embryos displayed precise genome editing across the majority of their cells (Fig. 4, Table S2?). Four embryos (T1, T2, T5 and T14) showed the presence of the edited allele at percentages between 96% and 100% indicating that all or close to all cells contain a 9 bp deletion as a biallelic modification. An additional three embryos (T6, T11 and T12) contained the edited allele at relatively high levels (between 71% and 88%) demonstrating that the majority of cells in these embryos carry the intended deletion. Again, we found embryos where the introgression was less efficient and contained the edited allele at levels ranging from 31% to 3%. Injection of RNA was similarly potent, with one (T16) out of four embryos displaying essentially complete conversion of the wild type alleles into edited alleles (Fig. 4, Table S2). Overall, the analysis of the logit-link model indicated that TALENs induce a better, though more variable rate of successful homology-directed repair than the ZFNs (p < 0.001). The model also indicated that the use of DNA-encoded nucleases gave better results than injections with RNA (p < 0.001). Furthermore, the interaction between nuclease and nucleic acid type was also highly significant (p < 0.001) with TALENs and DNA being the most efficient combination (Fig. S6B).

In some embryos we also observed the introduction of unintended mutations, most notably a 12 bp insertion in embryos Z4 and Z5 and inaccurate 2 bp, 9 bp and 4 bp deletions in embryos T9, T11 and T12 but also variants of the A and B wild type alleles that differ at the three polymorphic sites within the target region (Table S1B). All such variants with a representation of 1% or more are included in Table S1B while Fig. 4 only portrays the relative occurrence of alleles with intended edits and the two wild type alleles A and B.

## Discussion

Our initial experiments established that zygote injections of a ZFN pair designed to bind the bovine *LGB* locus result in targeted changes at the cleavage site. The chosen ZFN pair was designed against *LGB* isoform B and in their cell transfections, Yu *et al* determined a three times higher ZFN activity in cells containing two B alleles compared to those with two A alleles^10^. This preference is probably due to a SNP (Fig. 2) that may weaken the binding affinity of the 5’ ZFN monomer against isoform A. Contrary to this observation, we found that most of the indels were introduced into the A allele (Fig. 2, Table 2). Because of our use of abattoir-derived oocytes with unknown allelic composition, this bias may originate from a higher frequency of the A alleles in the oocytes that were used.

We initially co-injected GFP-encoding RNA as an indirect reporter for ZFN activity but there was no good correlation between GFP expression and genome-edited embryos; in some experiments, a large number of GFP-positive embryos proved unedited whereas absence of GFP fluorescence did not necessarily indicate a lack of ZFN expression (Table 1). In concordance with our observation, others have also reported only few GFP-positive porcine and bovine embryos following zygote injections^6^,^14^ suggesting that GFP expression, at least at the blastocyst stage, is a rather poor indicator for a successful injection.

NHEJ and homology-directed repair constitute competing pathways for the repair of double strand breaks, with NHEJ generally assumed to be the predominant repair mechanism in non-dividing cells whereas homology-directed repair is more frequent after DNA replication (reviewed in^15^). Moving on from NHEJ to homology-directed repair mediated by ODNs, we observed a drop in the number of precisely edited embryos as compared to the high frequency of up to 83% for NHEJ-triggered indel mutations that resulted from ZFN zygote injections without ODNs. However, both ZFNs and TALENs co-injections with ODN templates still produced between 11% and 46% of embryos with precisely edited alleles (Table 1). This compares favorably with published data from previous ZFN, TALENs and CRISPR/Cas9 zygote co-injections with DNA templates in mice, rats and rabbits^16^,^17^,^18^,^19^ although higher efficiencies were reported in a recent study with the generation of up to 95% biallelically-modified mice following CRISPR/Cas9/ODN co-injections^20^. Thus, our results demonstrate for the first time that homology-directed repair in ODN co-injected embryos facilitates highly efficient introgression of pre-designed genome alterations in an economically important livestock species.

Targeted mutations at a designer nuclease’s cut site can be introduced by either cytoplasmic or pronuclear zygote injections of both DNA and RNA^21^ and at different time points post fertilization. Injection of RNA is usually preferred since it can be readily translated into protein whereas plasmid DNA needs be transcribed first. Moreover, RNA-driven expression of genome-editing nucleases is temporarily limited while DNA can integrate into the genome which is not only unwanted from a regulatory point of view but may also result in undesired prolonged expression of the respective nuclease. We did not intend to perform pronuclear injections because the high lipid content of bovine oocytes obscures the pronuclei but tested cytoplasmic injections at two different injection times and delivery of RNA and DNA encoded designer nucleases. Deep sequence analysis of injected embryos revealed a high variability of the edited allele frequency for both time points. The identification of embryos with low to moderate counts for the edited allele indicates that both injection time points can generate embryos that contain some cells that have only monoallelic modifications or non-edited wild type cells. However, the generation of embryos with a 100% contribution of the edited allele following injection 18 h post fertilization demonstrates that the later injection time can produce biallelically modified, non-mosaic animals (Table 2, Table S2). Similarly, both DNA and RNA injections enabled the generation of fully converted embryos no longer containing the wild type allele. In spite of what appears to be an equivalent potential, the genome editing efficiency with DNA was in our hands significantly better than with RNA. Although embryo development with DNA and RNA injections was comparable, we have observed in subsequent experiments that RNA injections at 100 ng/μl can negatively affect embryo development dependent on the quality of the RNA preparation.

Zygote injections of designer nucleases like ZFNs and TALENs, either with or without ODN co-injections, are ultimately aimed at producing live animals after embryo transfer to recipient females. Given the long time period and high costs associated with producing genetically-modified livestock animals, such transfers are ideally limited to those embryos that carry biallelic modifications in almost all cells resulting in the birth of animals that readily display the editing-specified phenotypic trait and will transmit the edited genotype with high frequency. We were able to produce embryos with completely edited genomes by ZFN-mediated induction of indels and TALEN-mediated introgression of an ODN-specified, precise mutation. Approximately a quarter (5/19) of the TALENs/ODN co-injected embryos that were analyzed had a greater than 96% presence of the edited allele (Fig. 4, Table S2) making those ideal candidates for transfer and production of non-mosaic genome edited cattle. The efficiency of introgressing the same mutation with ZFNs was however much lower. Only one of the co-injected embryos showed a contribution of the edited allele of 61% while the contribution in all other embryos ranged between 22% and 0%. For the co-injections with TALENs, approximately 40% were screened positive for containing edited alleles. A quarter of those were shown to contain almost exclusively the edited allele which represents 10% of all injected embryos. Overall, comparing RNA- and DNA-encoded ZFNs and TALENs, the success rate for introgression of the template-encoded 9 bp deletion was highest with TALENs that were injected as DNA making it the most efficient combination in our study for the precise modification of this particular locus in the BLG gene. With the observed frequency of suitably edited blastocysts, it will be feasible to screen embryos prior to transfer using a small biopsy from the trophectoderm for genotyping. We have previously shown that this procedure does not compromise embryo development^22^ and will enable the selected transfer of validated embryos with high developmental competency.

As has been reported before^6^,^7^,^11^,^17^,^23^,^24^ we have also observed the occasional generation of embryos with heterozygous modifications, containing alleles with different indels such as embryo Z14 (Table S1). But the majority of the observed allelic variation is due to point mutations which are often represented as minor allelic variants (e.g. T14, Table S1). The positions showing the greatest sequence variation coincided with the three polymorphic sites for the wild type alleles A and B. Because these three sites were also polymorphic in the ODNs, new sequence combinations at these sites can be the result of independent editing events. Although all of the unintended mutations discussed above might have been caused by the nuclease activity of the injected ZFNs and TALENs, some may simply reflect sites that are polymorphic within the genetic pool of the used oocytes or could be derived from errors introduced during PCR amplification and sequencing.

Thus far, the generation of livestock animals with precisely introgressed genome alterations has been limited to SCNT-based strategies. Here, we show for the first time that highly efficient introgression in livestock can also be achieved through zygote injection of designer nucleases and ODN templates. We further demonstrate that TALENs/ODN co-injections produce fully converted, biallelically modified embryos which will enable the efficient generation of live cattle or other livestock, homozygous for a precisely edited allele. While we have formally only demonstrated this for ZFNs and TALENs one can expect that similar results will be achievable with the RNA-guided CRISPR/Cas9 system or novel editors that may become available in the near future.

## Materials and Methods

### *In vitro* embryo production

Collection of slaughterhouse ovaries, *in vitro* oocyte maturation (IVM) and IVF was carried out as described previously^25^,^26^. Prior to microinjection, the cumulus-corona of IVF zygotes was dispersed 8 h or 18 h post fertilization by vortexing oocytes in 500 μl of 1 mg/ml bovine testicular hyaluronidase in Hepes-buffered synthetic oviductal fluid (SOF).

### Site-specific nucleases

ZFN plasmids 25047/25048, previously described by Yu *et al.*^10^, were obtained from Sigma-Aldrich. The ZFN pair was designed for cleavage in exon 1 of the *LGB* gene variant B, 3’ of the ATG start codon. In some experiments, we used modified ZFN plasmids encoding fluorescently labeled ZFNs. 25047-FL and 25048-FL were obtained by inserting mCherry and EGFP cDNAs in frame with the ZFNs, respectively. Two TALEN pairs, btBLG1.1 and btBLG1.2, designed to target a region immediately downstream of the start codon of the bovine beta-lactoglobulin-encoding *LGB* gene (Fig. S5) were cloned into RCIscript TALEN plasmids as previously described^11^,^27^. Although these vectors are designed for *in vitro* transcription of TALEN mRNA, they showed excellent target-specific editing activity following their injection into bovine zygotes and were used for all TALEN injections with DNA.

### Cytoplasmic injection of DNA and RNA-encoded site-specific nucleases

All experiments were performed in accordance with the relevant guidelines and regulations and were approved by the New Zealand Environmental Protection Authority.

IVF zygotes were screened for extrusion of the second polar body or otherwise on the formation of pronuclei as indicators of fertilization. Suitable zygotes were cytoplasmically injected under an invert microscope (AX-70, Olympus) using a microinjection needle with an inner diameter of 4–5.5 μm (Vitrolife, USA). All nucleic acids were diluted in injection buffer (0.25 mM EDTA, 10 mM Tris-HCL pH 8.0) and approximately 10 pl of plasmid DNA (10 or 20 ng/μl for each of the two ZFNs or TALENs) or RNA (100 ng/μl, for each of the two ZFNs or TALENs) were delivered into the cytoplasm. For co-injections, the solutions contained an ODN (100 ng/μl) or GFP (50 ng/μl) in addition to ZFNs or TALENs. IVF zygotes were injected either 8 h or 18 h post fertilization, washed twice in HEPES-buffered SOF and kept in IVF medium prior to the *in vitro* culture (IVC) of embryos.

### Embryo culture

IVC was performed as described^28^. Briefly, injected embryos were co-cultured for 7 days (day 0: fertilization) in groups of ten embryos in 20 μl of early SOF medium with change over on D5 into late SOF media drops containing 10 μM 2, 4-dinitrophenol. All embryo cultures were kept under oil and atmospheric conditions of 5% CO_2_, 7% O_2_, and 88% N_2_. On day 7, embryos were assessed for their developmental stage as previously described^29^.

### *In vitro* transcription of RNA

ZFN RNA and TALEN RNA were synthesized with Ambion’s mMESSAGE mMACHINE T7 Ultra and T3 kits, respectively using XbaI-linearized ZFN and SacI-linearized TALEN plasmids as templates. Following poly-A-tailing of the capped transcript with Epicentre’s poly (A) polymerase tailing kit, the *in vitro* produced mRNA was purified with Qiagen’s RNeasy mini elute kit.

### Analysis of embryos

Morulae and blastocysts developed from injected zygotes were washed in PBS and lysed in 10 μl lysis buffer (50 mM Tris, pH 8.3, 10 mM KCl, 5 mM NH4(SO_4_)_2_, 2 mM MgCl_2_) with proteinase K (55 °C 15 min, 95 °C 15 min). Using the lysed embryos as PCR template, a 550 bp fragment of the *LGB* target locus, encompassing the centrally located ZFN and TALEN cut sites, was amplified with primers 840 and 841. Sequences of all primers used in this study are listed in Table S3. To enable detection of random NHEJ-triggered mutations at the ZFN cleavage site, 550 bp PCR amplicons were subcloned in pCR II TOPO (Life Technologies) and transformed into bacteria.

To analyze subcloned PCR fragments, part of the transformed bacterial colonies were directly transferred as template into the PCR reaction mixture of a real time PCR TaqMan assay (Corbett RG 6000: 95 °C 3 min followed by 40 × 95 °C 10 sec and 62 °C 25 sec, amplification primers 842 and 843 and 871 as TaqMan probe, Applied Biosystems). In addition, amplification products of individual subclones were analyzed for their melt temperatures following the final PCR cycle similar to the method of high resolution melt temperature analysis^30^. To determine the precise nature of the mutations (indels or SNPs) at the ZFN cleavage site, plasmid DNA was isolated from positively tested bacterial colonies for sequencing of the approximately 550 bp target site fragment.

Precise ODN-mediated mutations were detected by a nested, mutation specific PCR (ODN 970-injected embryos: primers 987 and 841, 60 °C annealing; ODN 986-injected embryos: primers 994 and 841, 68 °C annealing) with the 550 bp *LGB* fragment as template. To confirm the presence of the ODN-mediated mutations in selected embryos, 550 bp first PCR amplicons were subcloned into pCR II TOPO (ZFN-injected embryo samples) or pGEM Teasy (TALENs-injected embryo samples) and subjected to analytical restriction digests with either XbaI (ZFN/ODN 970-injected embryos), SfoI (ZFN/ODN 986-injected embryos) or SfoI/RsaI (TALENs/ ODN 970-injected embryos).

### Sequence analysis

Conventional sequencing of *LGB* fragments from individual subclones was performed by Massey Genome Service.

To prepare samples for next generation sequencing, lysed embryo samples or *LGB* amplicons, pre-amplified from embryos (primers 840 and 841), were used as PCR templates in combination with primers 1016 and 1017 to generate fragments that were compatible with the Illumina two step amplicon library preparation method. Following amplification, ~400 bp fragments were gel-purified using the NucleoSpin Gel and PCR clean-up kit (Machery-Nagel) and used for sequencing on an Illumina Miseq at NZ Genomics Limited. The total yield (minus PhiX sequences) was 8,553,428 2 × 250 bp paired end reads, giving 11,000 to 405,000 paired reads per sample. The reads were then trimmed to their longest contiguous segment for which quality scores are less than 0.01, using the software DynamicTrim from the SolexaQA package^31^ (http://solexaqa.sourceforge.net/). The reads that overlapped by at least 14 bp with 100% identity were then joined using the fastq-join tool from the ea-utils package^32^. Joined reads less than 250 bp were removed. The length-filtered joined reads were then imported into Geneious^33^ and mapped to the target region of the bovine genome using the “map to reference” function with medium sensitivity and allowed to iterate up to 5 times. Geneious was then used to evaluate percentages of key mutations. The region containing the key mutations (NCBI accession AC_000168.1 bases 103,301,735 to 103,301,805) was extracted and copied into a text file using Geneious. The characters representing gaps were removed from each sequence. Each unique sequence was then enumerated using a Perl script. Exact matches to the reference sequence and the alternative “A” variant were counted for each sample along with sequences representing over 1% of all paired reads.

### Statistical analysis

The genome editing data summarized in the table 1 and table S2 were converted into comma separated value files and loaded into R version 3.0.1^34^. Trellis plots (Fig. S6) were generated from the data using the “lattice” package version 0.20–15^35^. For table 1 Fisher’s exact tests were used to determine significance between different factors.

For the deep sequencing data summarized in supplementary Table S2, a binomial logit link model ((Number of Reads edited, Number of reads not edited) ~Nucleic Acid * Enzyme type, family = binomial (logit))) was used to find factors that influenced the proportion of canonically edited embryos. As there was no TALEN data at 8 h, all data for the 8 h time point were left out of the model.

## Additional Information

**How to cite this article**: Wei, J. *et al.* Efficient introgression of allelic variants by embryo-mediated editing of the bovine genome. *Sci. Rep.* **5**, 11735; doi: 10.1038/srep11735 (2015).

## Supplementary Material

Supplementary table S1

Supplementary Information

## Figures and Tables

**Figure 1:**
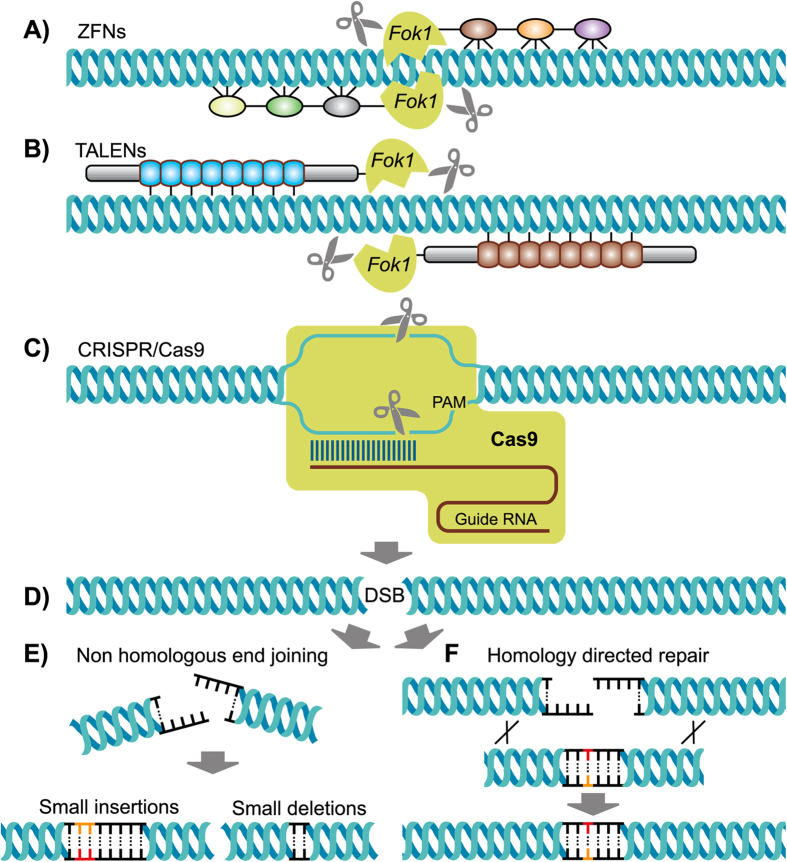
Schematic outline of the application of the three most commonly used genome editors (ZFNs, TALENs, CRISPRs) to introduce targeted genome modifications into animal genomes. ZFNs (**A**) are chimeric designer proteins that combine a customizable DNA binding domain with the catalytic domain of the restriction endonuclease FokI (green). The DNA binding domain is comprised of several, modular ZFs (colored ellipsoids) that each can bind to a specific triplet of DNA sequence. ZFNs require the coordinated binding of a pair of ZFNs at the target site to gain activity of the dimerization-dependent FokI nuclease. TALENs (**B**) are following the same principle but are using entirely different DNA binding modules (TALE repeats, blue and brown elements) that each specifically bind a single nucleotide. CRISPR/Cas9 (**C**) uses a universal nuclease with two catalytic domains (Cas9, green) that is guided to its specific target site by a stretch of 20 nucleotides of sequence complementarity of the guide RNA to the target sequence (indicated as base-pairing between the single stranded target DNA and guide RNA) in addition to the juxtaposition to the protospacer adjacent motif (PAM). All three editors (A to C) have in common that the targeted nuclease will cleave both DNA strands (indicated by the scissors) resulting in a double strand break (DSB) at the target site (**D**). This DSB can be repaired by the endogenous cellular machinery by non homologous end joining (**E**) which is an error prone mechanism leading to the frequent introduction of small insertions (colored bases) or deletions. If a homologous template is provided the DSB can be resolved through homology directed repair (**F**) allowing for the knockin of template-specified sequence variations (novel, colored base pair).

**Table 1:** Summary of *in vitro* embryo development and mutation rates observed in ZFN- and TALEN-injected IVF zygotes.

Reagent (ng/μl)	Protein	ODN	Zygotes injected	Time^a^	Blastocysts (%)	TM & BL^b^ (%)	Green (%)	Analyzed	Mutated (%)
DNA (20)	ZFN, GFP^c^		93	8 h	11/93 (12)		0/10 (0)	7	5/7 (71)
	ZFN, GFP^c^		89	18 h	10/89 (11)		0/10 (0)	6	5/6 (83)
RNA (100)	ZFN, GFP^c^		89	8 h	25/89 (28)		25/25 (100)	10	7/10 (70)
	ZFN, GFP^c^		90	18 h	33/90 (37)		33/33 (100)	7	2/7 (29)
DNA (20)	ZFN-FL^d^	970	110	8 h		11/110 (10)	ND^e^	11	2/11 (18)
	ZFN-FL^d^	970	252	18 h		37/252 (15)	ND^e^	18	6/18 (33)
	ZFN-FL^d^	986	99	8 h		16/99 (16)	ND^e^	12	4/12 (33)
	ZFN-FL^d^	986	89	18 h		17/89 (19)	ND^e^	15	4/15 (27)
RNA (100)	ZFN	986	139	8 h		17/139 (12)		17	2/17 (12)
	ZFN	986	110	18 h		10/110 (9)		10	4/10 (40)
DNA (10)	btBLG 1.1	986	50	18 h	22/50 (44)			5	2/5 (40)
DNA (20)	btBLG 1.2	986	269	18 h	84/269 (31)			48	22/48 (46)^f^
RNA (100)	btBLG 1.2	986	127	18 h	37/127 (29)			35	4/35 (11)^f^

^a^Time: injection time post-IVF.

^b^TM & BL: tight morulae and blastocysts.

^c^injected as RNA.

^d^fluorescently-labelled ZFNs.

^e^ND: not determined.

^f^significance level p < 0.001.

**Table 2:** Summary of indels and their frequency in ZFN-injected embryos.

Sample	Material	Time	Variant 1^a^	%	Variant 2>^a^	%	% WT (A + B)
Z14	RNA	8 h	24 bp DEL-A	71	8 bp DEL-B	29	0
Z15	RNA	8 h	4 bp DEL-A	83			16
Z16	RNA	8 h	4 bp DEL-B	54			39
Z17	RNA	8 h	1 bp INS-A	100			0
Z18	DNA	8 h	A-T TV-A	3			94
Z19	DNA	8 h	11 bp DEL-A	12			84
Z20	DNA	8 h	13 different variants with single point mutations-A			1-2 (each)	79
Z22	DNA	18 h	9 bp DEL-B	93	T-A TV, 9 bp DEL-B	7	0
Z24	DNA	18 h	A-G TS-A	2			84

^a^allelic variants with sequence differences other than the polymorphic sites of the wild type alleles. DEL: deletion; INS: insertion; TV: transversion; TS: transition; A: mutation in wild type allele A; B: mutation in wild type allele B.
