# Supplementary material for: Efficient introgression of allelic variants by embryo-mediated editing of the bovine genome
Source: Sci Rep. 2015 Jul 9;5:11735. doi: 10.1038/srep11735 (PMC4496724; doi:10.1038/srep11735)
Supplement: Supplementary Information [file srep11735-s2.pdf]

**Efficient introgression of allelic variants by embryo-mediated editing of the bovine genome**

Jingwei Wei, Stefan Wagner, Dan Lu, Paul Maclean, Daniel F. Carlson, Scott C. Fahrenkrug, Götz Laible

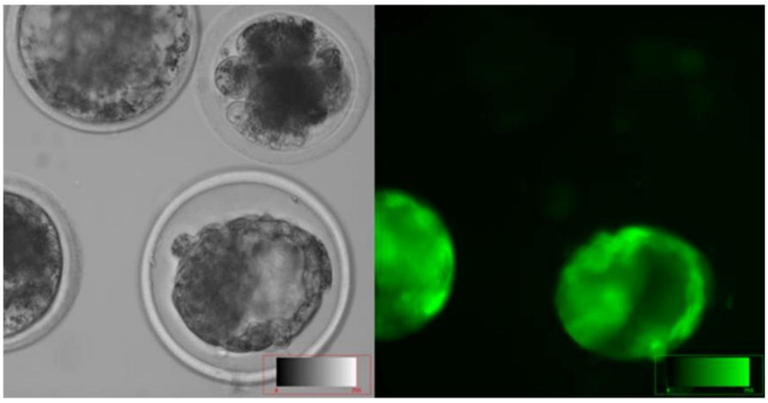

**Supplementary Fig. S1: GFP expression in embryos developed from zygotes injected with RNA encoding ZFNs and GFP.** Left panel: bright field; right panel: green fluorescence.

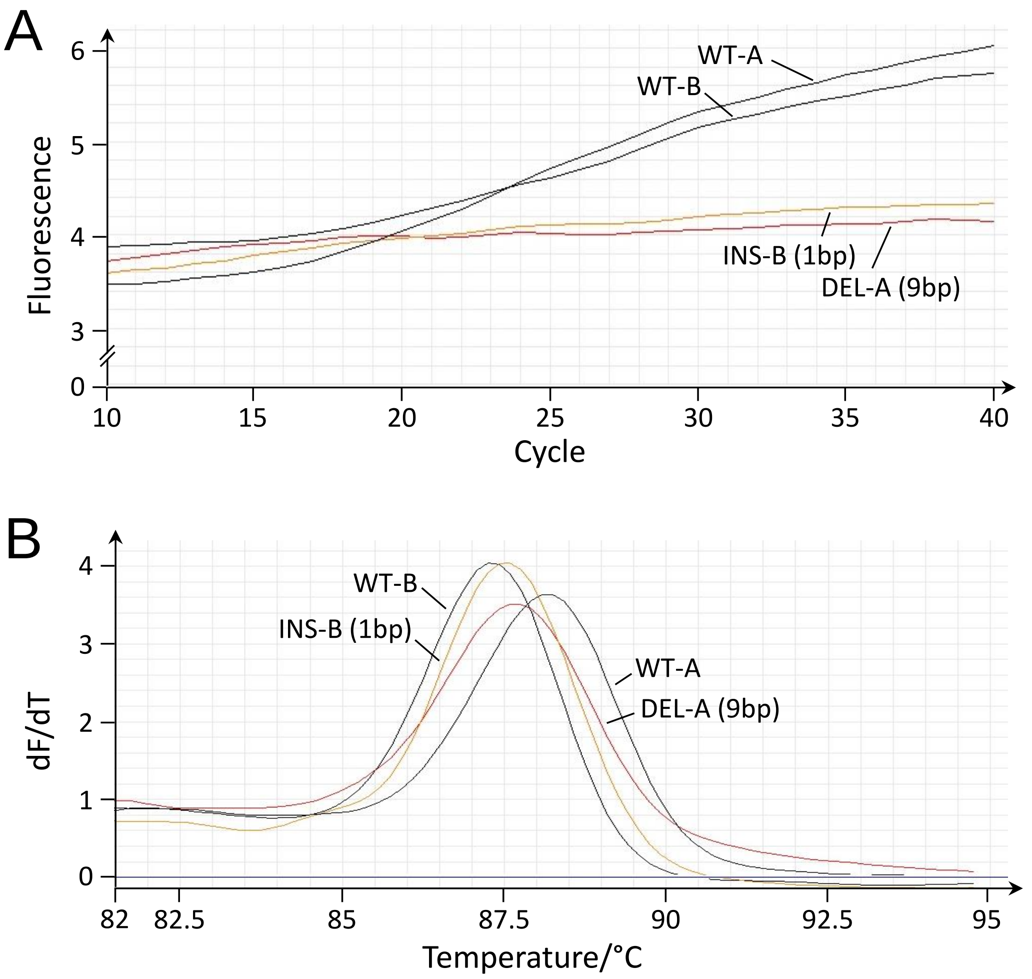


**Supplementary Fig. S2: Detection of random indels.** A) The fluorescent signal generated during PCR amplification (cycles 10-40) of two subclones containing the wild type sequence for variants A and B (WT-A, WT-B) and two subclones containing the variant B sequence with a 1 bp insertion (INS-B) and variant A sequence with a 9 bp deletion (DEL-A) using a TaqMan probe that is specific for the non-mutated wild type target sequence. B) Analysis of the melting temperature of the amplified PCR fragments from the same subclones.

```
...a  l  t  c  g  a  q  a  L  I  V  T  Q        T  M  L
...GCCCTCACYTGTGGCGCCCAGGCCCTCATYGTCACCC-----AGACCATGAAGGGCCTGGATATCCAGAAGGTTCGAGGGTGY...  WT allele
...|||||||||||||||||||||||||||||||||||||     ||||||||||||||||||||||||||||||||||||||
...GCCCTCACYTGTGGCGCCCAGGCCCTCATYGTCACCCtctagAGACCATGAAGGGCCTGGATATCCAGAAGGTTCGAGGG        ODN 970, 5 bp INS
...a  l  t  c  g  a  q  a  L  I  V  T  L  *
                                           XbaI

...a  l  t  c  g  a  q  a  L  I  V  T  Q  T  M  L  G  L
...GCCCTCACYTGTGGCGCCCAGGCCCTCATYGTCACCCAGACCATGAAGGGCCTGGCCTGGATATCCAGAAGGTTCGAGGGTGY...  WT allele
...||||||||||         ||||||||||||||||||||||||||||||||||||||||||||||||||||||||||||||||||
...GCCCTCACYT---------AGGCCCTCATYGTCACCCAGACCATGAAGGGCCTGGCCTGGATATCCAGAAGGTTCGAGGGTGY...  ODN 986, 9 bp DEL
...a  l  t  *
           SfoI
```

**Supplementary Fig. S3: Mutations specified by ODN 970 and ODN 986.** Comparison of relevant parts of WT and ODN *LGB* target sequence shows the nature of the ODN mutations and their relative position to the cleavage site (underlined) of the ZFN pair. The nucleotide Y (green highlight) specifies a pyrimidine base (C or T) at the site of the sequence polymorphism between the main two *LGB* variants A and B. The ODN 970-specified insertion (INS) is indicated by nucleotides in lower case and the deletion (DEL) for ODN 986 by hyphens. Newly created stop codons that disrupt the reading frame are highlighted in bold and restriction sites for XbaI and SfoI that differ in WT and ODN sequences are accentuated by grey boxes. The WT and ODN-encoded amino acids are given in single letter code with lower case indicating amino acids of the signal peptide and upper case amino acids of the mature BLG protein.

A) ZFN injection, DNA, ODN 970

```
Embryo 2    AGGCCTCCTATTGTCCTCGTAGAGGAAGCAACCCCAGGGCCCAAGGATAGGCCAGGGGGG   60
            ||||||||||||||||||||||||||||||||||||||||||||||||||||||||||||
WT B        AGGCCTCCTATTGTCCTCGTAGAGGAAGCAACCCCAGGGCCCAAGGATAGGCCAGGGGGG   60

Embryo 2    ATTCGGGGAACCGCGTGGCTGGGGGCCCGGCCCGGGCTGGCTGGCTGGCCCTCCTCCTGT  120
            ||||||||||||||||||||||||||||||||||||||||||||||||||||||||||||
WT B        ATTCGGGGAACCGCGTGGCTGGGGGCCCGGCCCGGGCTGGCTGGCTGGCCCTCCTCCTGT  120

Embryo 2    ATAAGGCCCCGAGCCCGCTGTCTCAGCCCTCCACTCCCTGCAGAGCTCAGAAGCGTGACC  180
            |||||||||||||||| |||||||||||||||||||||||||||||||||||||||||||
WT B        ATAAGGCCCCGAGCCCACTGTCTCAGCCCTCCACTCCCTGCAGAGCTCAGAAGCGTGACC  180

Embryo 2    CCAGCTGCAGCCATGAAGTGCCTCCTGCTTGCCCTGGCCCTCACTTGTGGCGCCCAGGCC  240
            ||||||||||||||||||||||||||||||||||||||||||||||||||||||||||||
WT B        CCAGCTGCAGCCATGAAGTGCCTCCTGCTTGCCCTGGCCCTCACTTGTGGCGCCCAGGCC  240

Embryo 2    CTCATTGTCACCCTCTAGAGACCATGAAGGGCCTGGATATCCAGAAGGTTCGAGGGTGCC  300
            |||||||||||||     ||||||||||||||||||||||||||||||||||||||||||
WT B        CTCATTGTCACCC-----AGACCATGAAGGGCCTGGATATCCAGAAGGTTCGAGGGTGCC  295

Embryo 2    CGGGTGGGTGGTGAGTTGCAGGGCAGGCAGGGGAGCTGGGCCTCAGAGACCAAGGGAGGC  360
            ||||||||||||||||||||||||||||||||||||||||||||||||||||||||||||
WT B        CGGGTGGGTGGTGAGTTGCAGGGCAGGCAGGGGAGCTGGGCCTCAGAGACCAAGGGAGGC  355

Embryo 2    TGTGACGTCTGGGATTCCCATCAGTCAGCTAGAGCCGCCTGACAAATCGCCCGCCAGGGC  420
            ||||||||||||||||||||||||||||||||||||||||||||||||||||||||||||
WT B        TGTGACGTCTGGGATTCCCATCAGTCAGCTAGAGCCGCCTGACAAATCGCCCGCCAGGGC  415

Embryo 2    AGCTTCAACCAGGCGTTTAGTGTCTTGCATTCTGGAGGCTGGAAGCCTGCAATCCGGGCA  480
            ||||||||||||||||||||||||||||||||||||||||||||||||||||||||||||
WT B        AGCTTCAACCAGGCGTTTAGTGTCTTGCATTCTGGAGGCTGGAAGCCTGCAATCCGGGCA  475

Embryo 2    TCGGCCCAGCTGGCTTCTCCTGCGGCCACTCTCCGGGGAGCAGACAGCCATCTTCTCCCT  540
            ||||||||||||||||||||||||||||||||||||||||||||||||||||||||||||
WT B        TCGGCCCAGCTGGCTTCTCCTGCGGCCACTCTCCGGGGAGCAGACAGCCATCTTCTCCCT  535

Embryo 2    GTGTCCTTTGC  551
            |||||||||||
WT B        GTGTCCTTTGC  546


Embryo 3    AGGCCTCCTATTGTCCTCGTAGAGGAAGCAACCCCAGGGCCCAAGGATAGGCCAGGGGGG   60
            ||||||||||||||||||||||||||||||||||||||||||||||||||||||||||||
WT B        AGGCCTCCTATTGTCCTCGTAGAGGAAGCAACCCCAGGGCCCAAGGATAGGCCAGGGGGG   60

Embryo 3    ATTCGGGGAACCGCGTGGCTGGGGGCCCGGCCCGGGCTGGCTGGCTGGCCCTCCTCCTGT  120
            ||||||||||||||||||||||||||||||||||||||||||||||||||||||||||||
WT B        ATTCGGGGAACCGCGTGGCTGGGGGCCCGGCCCGGGCTGGCTGGCTGGCCCTCCTCCTGT  120

Embryo 3    ATAAGGCCCCGAGCCCGCTGTCTCAGCCCTCCACTCCCTGCAGAGCTCAGAAGCGTGACC  180
            |||||||||||||||| |||||||||||||||||||||||||||||||||||||||||||
WT B        ATAAGGCCCCGAGCCCACTGTCTCAGCCCTCCACTCCCTGCAGAGCTCAGAAGCGTGACC  180

Embryo 3    CCAGCTGCAGCCATGAAGTGCCTCCTGCTTGCCCTGGCCCTCACTTGTGGCGCCCAGGCC  240
            ||||||||||||||||||||||||||||||||||||||||||||||||||||||||||||
WT B        CCAGCTGCAGCCATGAAGTGCCTCCTGCTTGCCCTGGCCCTCACTTGTGGCGCCCAGGCC  240
```

```
Embryo 3    CTCGTCGTCACCCTCTAGAGACCATGAAGGGCCTGGATATCCAGAAGGTTCGAGGGTGCC  300
            ||| | |||||||     ||||||||||||||||||||||||||||||||||||||||||
WT B        CTCATTGTCACCC-----AGACCATGAAGGGCCTGGATATCCAGAAGGTTCGAGGGTGCC  295

Embryo 3    CGGGTGGGTGGTGAGTTGCAGGGCAGGCAGGGGAGCTGGGCCTCAGAGACCAAGGGAGGC  360
            ||||||||||||||||||||||||||||||||||||||||||||||||||||||||||||
WT B        CGGGTGGGTGGTGAGTTGCAGGGCAGGCAGGGGAGCTGGGCCTCAGAGACCAAGGGAGGC  355

Embryo 3    TGTGACGTCTGGGATTCCCATCAGTCAGCTAGAGCCGCCTGACAAATCGCCCGCCAGGGC  420
            ||||||||||||||||||||||||||||||||||||||||||||||||||||||||||||
WT B        TGTGACGTCTGGGATTCCCATCAGTCAGCTAGAGCCGCCTGACAAATCGCCCGCCAGGGC  415

Embryo 3    AGCTTCAACCAGGCGTTTAGTGTCTTGCATTCTGGAGGCTGGAAGCCTGCAATCCGGGCA  480
            ||||||||||||||||||||||||||||||||||||||||||||||||||||||||||||
WT B        AGCTTCAACCAGGCGTTTAGTGTCTTGCATTCTGGAGGCTGGAAGCCTGCAATCCGGGCA  475

Embryo 3    TCGGCCCAGCTGGCTTCTCCTGCGGCCACTCTCCGGGGAGCAGACAGCCATCTTCTCCCT  540
            ||||||||||||||||||||||||||||||||||||||||||||||||||||||||||||
WT B        TCGGCCCAGCTGGCTTCTCCTGCGGCCACTCTCCGGGGAGCAGACAGCCATCTTCTCCCT  535

Embryo 3    GTGTCCTTTGC  551
            |||||||||||
WT B        GTGTCCTTTGC  546


Embryo 6    AGGCCTCCTATTGTCCTCGTAGAGGAAGCAACCCCAGGGCCCAAGGATAGGCCAGGGGGG   60
            ||||||||||||||||||||||||||||||||||||||||||||||||||||||||||||
WT B        AGGCCTCCTATTGTCCTCGTAGAGGAAGCAACCCCAGGGCCCAAGGATAGGCCAGGGGGG   60

Embryo 6    ATTCGGGGAACCGCGTGGCTGGGGGCCCGGCCCGGGCTGGCTGGCTGGCCCTCCTCCTGT  120
            ||||||||||||||||||||||||||||||||||||||||||||||||||||||||||||
WT B        ATTCGGGGAACCGCGTGGCTGGGGGCCCGGCCCGGGCTGGCTGGCTGGCCCTCCTCCTGT  120

Embryo 6    ATAAGGCCCCGAGCCCGCTGTCTCAGCCCTCCACTCCCTGCAGAGCTCAGAAGCGTGACC  180
            |||||||||||||||| |||||||||||||||||||||||||||||||||||||||||||
WT B        ATAAGGCCCCGAGCCCACTGTCTCAGCCCTCCACTCCCTGCAGAGCTCAGAAGCGTGACC  180

Embryo 6    CCAGCTGCAGCCATGAAGTGCCTCCTGCTTGCCCTGGCCCTCACTTGTGGCGCCCAGGCC  240
            ||||||||||||||||||||||||||||||||||||||||||||||||||||||||||||
WT B        CCAGCTGCAGCCATGAAGTGCCTCCTGCTTGCCCTGGCCCTCACTTGTGGCGCCCAGGCC  240

Embryo 6    CTCATTGTCACCCTCTAGAGACCATGAAGGGCCTGGATATCCAGAAGGTTCGAGGGTGCC  300
            |||||||||||||     ||||||||||||||||||||||||||||||||||||||||||
WT B        CTCATTGTCACCC-----AGACCATGAAGGGCCTGGATATCCAGAAGGTTCGAGGGTGCC  295

Embryo 6    CGGGTGGGTGGTGAGTTGCAGGGCAGGCAGGGGAGCTGGGCCTCAGAGACCAAGGGAGGC  360
            ||||||||||||||||||||||||||||||||||||||||||||||||||||||||||||
WT B        CGGGTGGGTGGTGAGTTGCAGGGCAGGCAGGGGAGCTGGGCCTCAGAGACCAAGGGAGGC  355

Embryo 6    TGTGACGTCTGGGATTCCCATCAGTCAGCTAGAGCCGCCTGACAAATCGCCCGCCAGGGC  420
            ||||||||||||||||||||||||||||||||||||||||||||||||||||||||||||
WT B        TGTGACGTCTGGGATTCCCATCAGTCAGCTAGAGCCGCCTGACAAATCGCCCGCCAGGGC  415

Embryo 6    AGCTTCAACCAGGCGTTTAGTGTCTTGCATTCTGGAGGCTGGAAGCCTGCAATCCGGGCA  480
            ||||||||||||||||||||||||||||||||||||||||||||||||||||||||||||
WT B        AGCTTCAACCAGGCGTTTAGTGTCTTGCATTCTGGAGGCTGGAAGCCTGCAATCCGGGCA  475

Embryo 6    TCGGCCCAGCTGGCTTCTCCTGCGGCCACTCTCCGGGGAGCAGACAGCCATCTTCTCCCT  540
            ||||||||||||||||||||||||||||||||||||||||||||||||||||||||||||
WT B        TCGGCCCAGCTGGCTTCTCCTGCGGCCACTCTCCGGGGAGCAGACAGCCATCTTCTCCCT  535
```

```
Embryo 6    GTGTCCTTTGC   551
            |||||||||||
WT B        GTGTCCTTTGC   546
```

B) ZFN injection, DNA, ODN 986

```
Embryo 1    AGGCCTCYTATTGTCCTMGTAGAGGAAGCAACCCCAGGGCCCAAGGATAGGCCAGGGGGG    60
            ||||||| ||||||||| ||||||||||||||||||||||||||||||||||||||||||
WT B        AGGCCTCCTATTGTCCTCGTAGAGGAAGCAACCCCAGGGCCCAAGGATAGGCCAGGGGGG    60

Embryo 1    ATTCGGGGAACCGCGTGGCTGGGGGCCCGGCCCGGGCTGGCTGGCTGGCCCTCCTCCTGT   120
            ||||||||||||||||||||||||||||||||||||||||||||||||||||||||||||
WT B        ATTCGGGGAACCGCGTGGCTGGGGGCCCGGCCCGGGCTGGCTGGCTGGCCCTCCTCCTGT   120

Embryo 1    ATAAGGCCCCGAGCCCGCTGTCTCAGCCCTCCACTCCYTGCAGAGCTCAGAAGCGTGACC   180
            |||||||||||||||| |||||||||||||||||||| ||||||||||||||||||||||
WT-B        ATAAGGCCCCGAGCCCACTGTCTCAGCCCTCCACTCCCTGCAGAGCTCAGAAGCGTGACC   180

Embryo 1    CCAGCTGCAGCCATGAAGTGCCTCCTGCTTGCCCTGGCCCTCACTT---------AGGCC   231
            ||||||||||||||||||||||||||||||||||||||||||||||         |||||
WT-B        CCAGCTGCAGCCATGAAGTGCCTCCTGCTTGCCCTGGCCCTCACTTGTGGCGCCCAGGCC   240

Embryo 1    CTCATTGTCACCCAGACCATGAAGGGCCTGGATATCCAGAAGGTTCGAGGGTGCCCGGGT   291
            ||||||||||||||||||||||||||||||||||||||||||||||||||||||||||||
WT-B        CTCATTGTCACCCAGACCATGAAGGGCCTGGATATCCAGAAGGTTCGAGGGTGCCCGGGT   300

Embryo 1    GGGTGGTGAGTTGCAGGGCAGGCAGGGGAGCTGGGCCTCAGAGACCAAGGGAGGCTGTGA   351
            ||||||||||||||||||||||||||||||||||||||||||||||||||||||||||||
WT-B        GGGTGGTGAGTTGCAGGGCAGGCAGGGGAGCTGGGCCTCAGAGACCAAGGGAGGCTGTGA   360

Embryo 1    CGTCTGGGATTCCCATCAGTCAGCTAGAGCCGCCTGACAAATCGCCCGCCAGGGCAGCTT   411
            ||||||||||||||||||||||||||||||||||||||||||||||||||||||||||||
WT-B        CGTCTGGGATTCCCATCAGTCAGCTAGAGCCGCCTGACAAATCGCCCGCCAGGGCAGCTT   420

Embryo 1    CAACCAGGCGTTTAGTGTCTTGCATTCTGGAGGCTGGAAGCCTGCAATCCGGGCATCGGC   471
            ||||||||||||||||||||||||||||||||||||||||||||||||||||||||||||
WT-B        CAACCAGGCGTTTAGTGTCTTGCATTCTGGAGGCTGGAAGCCTGCAATCCGGGCATCGGC   480

Embryo 1    CCAGCTGGCTTCTCCTGCGGCCACTCTCCGGGGAGCAGACAGCCATCTTCTCCCTGTGTC   531
            ||||||||||||||||||||||||||||||||||||||||||||||||||||||||||||
WT-B        CCAGCTGGCTTCTCCTGCGGCCACTCTCCGGGGAGCAGACAGCCATCTTCTCCCTGTGTC   540

Embryo 1    CTTTGC   537
            ||||||
WT-B        CTTTGC   546


Embryo 2    AGGCCTCCTATTGTCCTCGTAGAGGAAGCAACC---------------------------    33
            |||||||||||||||||||||||||||||||||
WT B        AGGCCTCCTATTGTCCTCGTAGAGGAAGCAACCCCAGGGCCCAAGGATAGGCCAGGGGGG    60

Embryo 2    ------------------------------------------------------------    33

WT B        ATTCGGGGAACCGCGTGGCTGGGGGCCCGGCCCGGGCTGGCTGGCTGGCCCTCCTCCTGT   120

Embryo 2    ------------------------------------------------------------    33

WT-B        ATAAGGCCCCGAGCCCACTGTCTCAGCCCTCCACTCCCTGCAGAGCTCAGAAGCGTGACC   180
```

```
Embryo 2    --------------------CCTCATGCTTGCCCTGGCCCTCACTT---------AGGCC   64
                                |||| |||||||||||||||||||||         |||||
WT-B        CCAGCTGCAGCCATGAAGTGCCTCCTGCTTGCCCTGGCCCTCACTTGTGGCGCCCAGGCC  240

Embryo 2    CTCATCGTCACCCAGACCATGAAGGGCCTGGATATCCAGAAGGTTCGAGGGTGCCCGGGT  124
            ||||| ||||||||||||||||||||||||||||||||||||||||||||||||||||||
WT-B        CTCATTGTCACCCAGACCATGAAGGGCCTGGATATCCAGAAGGTTCGAGGGTGCCCGGGT  300

Embryo 2    GGGTGGTGAGTTGCAGGGCAGGCAGGGGAGCTGGGCCTCAGAGACCAAGGGAGGCTGTGA  184
            ||||||||||||||||||||||||||||||||||||||||||||||||||||||||||||
WT-B        GGGTGGTGAGTTGCAGGGCAGGCAGGGGAGCTGGGCCTCAGAGACCAAGGGAGGCTGTGA  360

Embryo 2    CGTCTGGGATTCCCATCAGTCAGCTAGAGCCGCCTGACAAATCGCCCGCCAGGGCAGCTT  244
            ||||||||||||||||||||||||||||||||||||||||||||||||||||||||||||
WT-B        CGTCTGGGATTCCCATCAGTCAGCTAGAGCCGCCTGACAAATCGCCCGCCAGGGCAGCTT  420

Embryo 2    CAACCAGGCGTTTAGTGTCTTGCATTCTGGAGGCTGGAAGCCTGCAATCCGGGCATCGGC  304
            ||||||||||||||||||||||||||||||||||||||||||||||||||||||||||||
WT-B        CAACCAGGCGTTTAGTGTCTTGCATTCTGGAGGCTGGAAGCCTGCAATCCGGGCATCGGC  480

Embryo 2    CCAGCTGGCTTCTCCTGCGGCCACTCTCCGGGGAGCAGACAGCCATCTTCTCCCTGTGTC  364
            ||||||||||||||||||||||||||||||||||||||||||||||||||||||||||||
WT-B        CCAGCTGGCTTCTCCTGCGGCCACTCTCCGGGGAGCAGACAGCCATCTTCTCCCTGTGTC  540

Embryo 2    CTTTGC  370
            ||||||
WT-B        CTTTGC  546


Embryo 3    AGGCCTCCTATTGTCCTCGTAGAGGAAGCAACCCCAGGGCCCAAGGATACGCCAGGGGGG   60
            ||||||||||||||||||||||||||||||||||||||||||||||||| ||||||||||
WT A        AGGCCTCCTATTGTCCTCGTAGAGGAAGCAACCCCAGGGCCCAAGGATAGGCCAGGGGGG   60

Embryo 3    ATTCGGGGAACCGCGTGGCTGGGGGCCCGGCCCGGGCTGGCTGGCTGGCCCTCCTCCTGT  120
            ||||||||||||||||||||||||||||||||||||||||||||||||||||||||||||
WT A        ATTCGGGGAACCGCGTGGCTGGGGGCCCGGCCCGGGCTGGCTGGCTGGCCCTCCTCCTGT  120

Embryo 3    ATAAGGCCCCGAGCCCGCTGTCTCAGCCCTCCACTCCCTGCAGAGCTCAGAAGCGTGATC  180
            |||||||||||||||| |||||||||||||||||||||||||||||||||||||||| |
WT A        ATAAGGCCCCGAGCCCACTGTCTCAGCCCTCCACTCCCTGCAGAGCTCAGAAGCGTGACC  180

Embryo 3    CCGGCTGCAGCCATGAAGTGCCTCCTGCTTGCCCTGGCCCTCACTT---------AGGCC  231
            || ||||||||||||||||||||||||||||||||||||||||| |         |||||
WT A        CCAGCTGCAGCCATGAAGTGCCTCCTGCTTGCCCTGGCCCTCACCTGTGGCGCCCAGGCC  240

Embryo 3    CTCATCCTCACCCAGACCATGAAGGGCCTGGATATCCAGAAGGTTCGAGGGTGGCCGGGT  291
            |||||| |||||||||||||||||||||||||||||||||||||||||||||||||||||
WT A        CTCATCGTCACCCAGACCATGAAGGGCCTGGATATCCAGAAGGTTCGAGGGTGGCCGGGT  300

Embryo 3    GGGTGGTGAGTTGCAGGGCAGGCAGGGGAGCTGGGCCTCAGAGACCAAGGGAGGCTGTGA  351
            ||||||||||||||||||||||||||||||||||||||||||||||||||||||||||||
WT A        GGGTGGTGAGTTGCAGGGCAGGCAGGGGAGCTGGGCCTCAGAGACCAAGGGAGGCTGTGA  360

Embryo 3    CGTCTGGGATTCCCATCAGTCAGCTAGAGCCGCCTGACAAATCGCCCGCCAGGGCAGCTT  411
            ||||||||||||||||||||||||||||||||||||||||||||||||||||||||||||
WT A        CGTCTGGGATTCCCATCAGTCAGCTAGAGCCGCCTGACAAATCGCCCGCCAGGGCAGCTT  420

Embryo 3    CAACCAGGCGTTTAGTGTCTTGCATTCTGGAGGCTGGAAGCCTGCAATCCAGGCATCGGC  471
            |||||||||||||||||||||||||||||||||||||||||||||||||| |||||||||
WT A        CAACCAGGCGTTTAGTGTCTTGCATTCTGGAGGCTGGAAGCCTGCAATCCGGGCATCGGC  480
```

```
Embryo 3    CCAGCTGGCTTCTCCTGCGGCCACTCTCCGGGGAGCAGACAGCCATCTTCTCCCTGTGTC  531
            ||||||||||||||||||||||||||||||||||||||||||||||||||||||||||||
WT A        CCAGCTGGCTTCTCCTGCGGCCACTCTCCGGGGAGCAGACAGCCATCTTCTCCCTGTGTC  540

Embryo 3    CTTTGC  537
            ||||||
WT A        CTTTGC  546
```

C) TALEN injection, DNA, ODN 986

```
Embryo 1    AGGCCTCCTATTGTCCTCGTAGAGGAAGCAACCCCAGGGCCCAAGGATAGGCCAGGGGGG   60
            ||||||||||||||||||||||||||||||||||||||||||||||||||||||||||||
WT-B        AGGCCTCCTATTGTCCTCGTAGAGGAAGCAACCCCAGGGCCCAAGGATAGGCCAGGGGGG   60

Embryo 1    ATTCGGGGAACCGCGTGGCTGGGGGCCCGGCCCGGGCTGGCTGGCTGGCCCTCCTCCTGT  120
            ||||||||||||||||||||||||||||||||||||||||||||||||||||||||||||
WT-B        ATTCGGGGAACCGCGTGGCTGGGGGCCCGGCCCGGGCTGGCTGGCTGGCCCTCCTCCTGT  120

Embryo 1    ATAAGGCCCCGAGCCCGCTGTCTCAGCCCTCCACTCCCTGCAGAGCTCAGAAGCGTGACC  180
            |||||||||||||||| |||||||||||||||||||||||||||||||||||||||||||
WT-B        ATAAGGCCCCGAGCCCACTGTCTCAGCCCTCCACTCCCTGCAGAGCTCAGAAGCGTGACC  180

Embryo 1    CCAGCTGCAGCCATGAAGTGCCTCCTGCTTGCCCTGGCCCTCACTT---------AGGCC  231
            ||||||||||||||||||||||||||||||||||||||||||||||         |||||
WT-B        CCAGCTGCAGCCATGAAGTGCCTCCTGCTTGCCCTGGCCCTCACTTGTGGCGCCCAGGCC  240

Embryo 1    CTCATCGTCACCCAGACCATGAAGGGCCTGGATATCCAGAAGGTTCGAGGGTGCCCGGGT  291
            ||||| ||||||||||||||||||||||||||||||||||||||||||||||||||||||
WT-B        CTCATTGTCACCCAGACCATGAAGGGCCTGGATATCCAGAAGGTTCGAGGGTGCCCGGGT  300

Embryo 1    GGGTGGTGAGTTGCAGGGCAGGCAGGGGAGCTGGGCCTCAGAGACCAAGGGAGGCTGTGA  351
            ||||||||||||||||||||||||||||||||||||||||||||||||||||||||||||
WT-B        GGGTGGTGAGTTGCAGGGCAGGCAGGGGAGCTGGGCCTCAGAGACCAAGGGAGGCTGTGA  360

Embryo 1    CGTCTGGGATTCCCATCAGTCAGCTAGAGCCGCCTGACAAATCGCCCGCCAGGGCAGCTT  411
            ||||||||||||||||||||||||||||||||||||||||||||||||||||||||||||
WT-B        CGTCTGGGATTCCCATCAGTCAGCTAGAGCCGCCTGACAAATCGCCCGCCAGGGCAGCTT  420

Embryo 1    CAACCAGGCGTTTAGTGTCTTGCATTCTGGAGGCTGGAAGCCTGCAATCCGGGCATCGGC  471
            ||||||||||||||||||||||||||||||||||||||||||||||||||||||||||||
WT-B        CAACCAGGCGTTTAGTGTCTTGCATTCTGGAGGCTGGAAGCCTGCAATCCGGGCATCGGC  480

Embryo 1    CCAGCTGGCTTCTCCTGCGGCCACTCTCCGGGGAGCAGACAGCCATCTTCTCCCTGTGTC  531
            ||||||||||||||||||||||||||||||||||||||||||||||||||||||||||||
WT-B        CCAGCTGGCTTCTCCTGCGGCCACTCTCCGGGGAGCAGACAGCCATCTTCTCCCTGTGTC  540

Embryo 1    CTTTGC  537
            ||||||
WT-B        CTTTGC  546
```

TALEN injection, RNA, ODN 986

```
Embryo 1    GGCTGGCCCTCCTCCTGTATAAGGCCCCGAGCCCGCTGTCTCAGCCCTCCACTCCCTGCA   60
            ||||||||||||||||||||||||||||||||||||||||||||||||||||||||||||
WT-A        GGCTGGCCCTCCTCCTGTATAAGGCCCCGAGCCCGCTGTCTCAGCCCTCCACTCCCTGCA   60
```

```
Embryo 1    GAGCTCAGAAGCGTGATCCCGGCTGCAGCCATGAAGTGCCTCCTGCTTGCCCTGGCCCTC  120
            |||||||||||||||| |||||||||||||||||||||||||||||||||||||||||||
WT-A        GAGCTCAGAAGCGTGACCCCGGCTGCAGCCATGAAGTGCCTCCTGCTTGCCCTGGCCCTC  120

Embryo 1    ACTT---------AGGCCCTCATCGTCACCCAGACCATGAATGGCCTGGATATCCAGAAG  171
            || |         |||||||||||||||||||||||||||| ||||||||||||||||||
WT-A        ACCTGTGGCGCCCAGGCCCTCATCGTCACCCAGACCATGAAGGGCCTGGATATCCAGAAG  180

Embryo 1    GTTCGAGGGTGGCCGGGTGGGTGGTGAGTTGCAGGGCAGGCAGGGGAGCTGGGCCTCAGA  231
            ||||||||||||||||||||||||||||||||||||||||||||||||||||||||||||
WT-A        GTTCGAGGGTGGCCGGGTGGGTGGTGAGTTGCAGGGCAGGCAGGGGAGCTGGGCCTCAGA  240

Embryo 1    GACCAAGGGAGGCTGTGACGTCTGGGATTCCCATCAGTCAGCTAGAGCCGCCTGACAAAT  291
            ||||||||||||||||||||||||||||||||||||||||||||||||||||||||||||
WT-A        GACCAAGGGAGGCTGTGACGTCTGGGATTCCCATCAGTCAGCTAGAGCCGCCTGACAAAT  300

Embryo 1    CGCCCGCCAGGGCAGCTTCAACCAGGCGTTTAGTGTCTTGC  392
            |||||||||||||||||||||||||||||||||||||||||
WT-A        CGCCCGCCAGGGCAGCTTCAACCAGGCGTTTAGTGTCTTGC  401
```

**Supplementary Fig. S4: Sequence of alleles with ODN-defined genome edits.** Alignment of the relevant parts of the *LGB* target sequence of subclones derived from individual embryos co-injected with ZFN-DNA/ODN 970 (A), ZFN-DNA/ODN 986 (B) and TALEN (btBLG1.2)-DNA (C) and TALEN (btBLG1.2)-RNA/ODN 986 (D) with the wild type sequence. ODN-specified 5 bp insertions (ODN 970) and 9 bp deletions (ODN 986) are highlighted in yellow. Nucleotides highlighted in green depict the three polymorphic sites between wild type alleles A and B which are also polymorphic in the ODNs that were used as repair templates. Any mutations unrelated to the ODN templates are shown in red. Hyphens indicate deletions and the newly generated stop codon TAG is underlined. The numbering refers to relative positions within the *LGB* sequence. Y: C or T; M: A or C.

A)

| | | |
|---|---|---|
| 18 | NH NI NI NH NG NH HD HD NG HD HD NG NH HD NG NG NH HD | 5’ btBLG 1.1 |
| 15 | NH NI NH NH NH HD HD NG NH NH NH HD NH HD HD | 3’ btBLG 1.1 |
| 17 | HD HD NG NH HD NG NG NH HD HD HD NG NH NH HD HD HD | 5’ btBLG 1.2 |
| 19 | HD NG NH NH NH NG NH NI HD NI NI NG NH NI NH NH NH HD HD | 3’ btBLG 1.2 |

B)

```
         m  k  c  l  l  l  a  l  a  l  t  c  g  a  q  a  L  I  V  T  Q  T  M...
...tgcagccATGAAGTGCCTCCTGCTTGCCCTGGCCCTCACYTGTGGCGCCCAGGCCCTCATYGTCACCCAGACCATG...  btBLG 1.1
...tgcagccATGAAGTGCCTCCTGCTTGCCCTGGCCCTCACYTGTGGCGCCCAGGCCCTCATYGTCACCCAGACCATG...  btBLG 1.2
...tgcagccATGAAGTGCCTCCTGCTTGCCCTGGCCCTCACYT---------AGGCCCTCATYGTCACCCAGACCATG...  ODN 986
         m  k  c  l  l  l  a  l  a  l  t  *
```

**Supplementary Fig. S5: Characteristics of the BLG-TALEN’s DNA binding domains and binding sites.** A) Summary of the number and identity of the repeat variable di-residue codes for each of the 5’ and 3’ monomers of the TALEN pairs btBLG 1.1 and btBLG 1.2 with specificity for the Bos taurus BLG gene. B) Shown are the binding sites (grey boxes) of the TALEN pairs btBLG 1.1 and btBLG 1.2 relative to the DNA sequence of the *LGB* target locus. Relevant parts of the ODN 986 sequence illustrate the location of the 9 bp that are missing in the ODN 986 sequence. Non-coding DNA sequences are indicated by lower case, coding sequences by upper case. The nucleotide Y (green) specifies a pyrimidine base (C or T) at polymorphic sequences between the main two *LGB* variants A and B and the underlined sequence shows the presence of a Sfo I restriction site. The ATG start codon is highlighted (bold) and the encoded amino acids of BLG are given in single letter code above the DNA sequence with lower case depicting amino acids of the signal peptide and upper case amino acids of the mature protein. The single letter amino acid code below the ODN 986 sequence shows the premature termination of the reading frame caused by the 9 bp deletion.

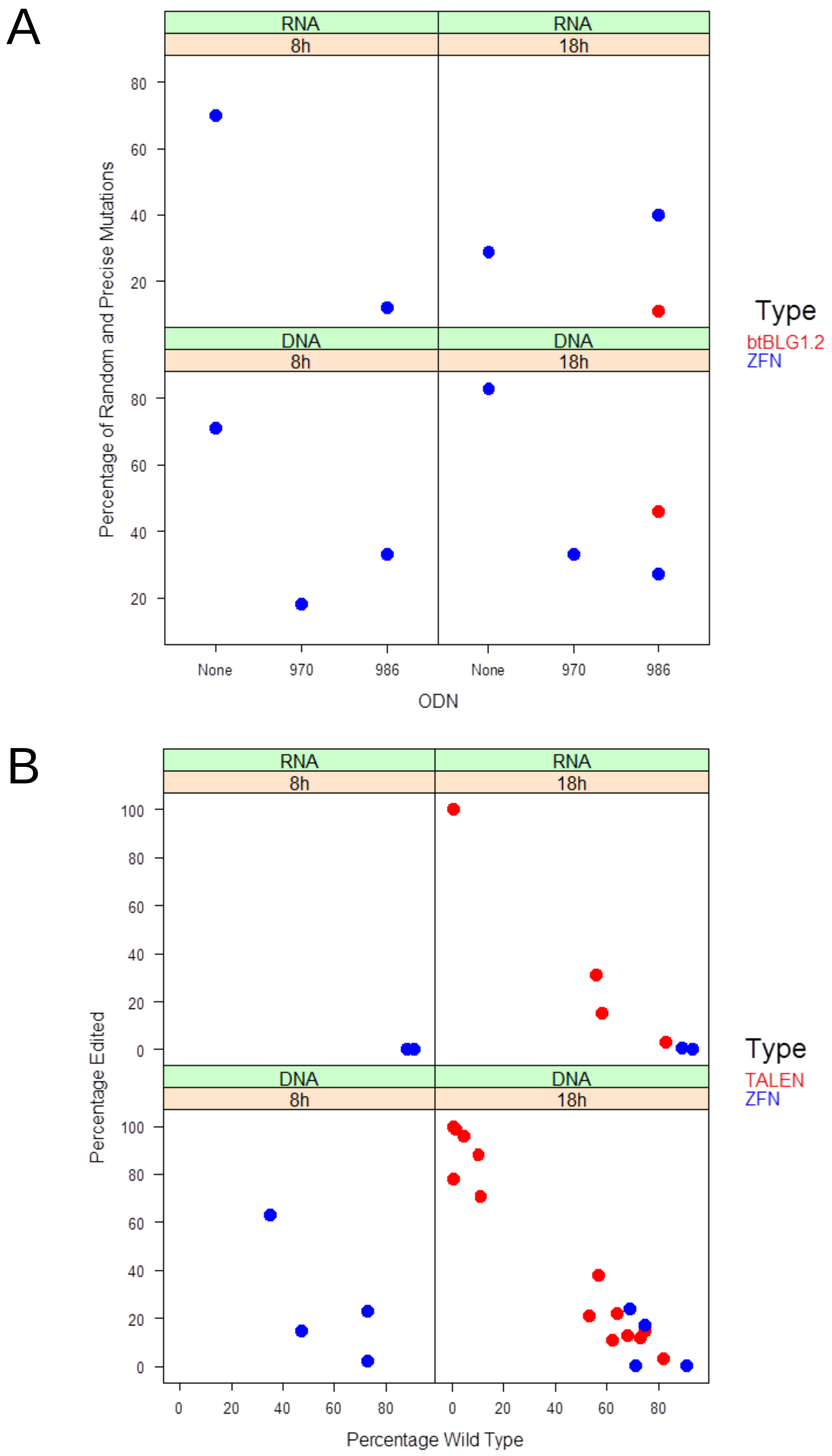


**Supplementary Fig. S6: Trellis plots of Tables 1 and S2.** A) Plot of percentage of random and precise mutations and ODN. The percentage of both random and precise mutations were combined and plotted against the levels of ODN, split by time and material (RNA or DNA). The points on the graph represent the genome editing results for individual embryos and are

coloured according to the application of ZFNs or TALENs (btBLG1.2). The underlying values used to generate the graph can be found in Table 1. B) Plot of percentage edited and percentage wild type alleles. The percentages of deep sequencing reads containing wild type and those containing canonically edited sequence were plotted, split by time and material. The points on the graph represent the genome editing results for individual embryos and are colored according to the application of ZFNs or TALENs. The underlying values used to generate the graph can be found in Table S2.

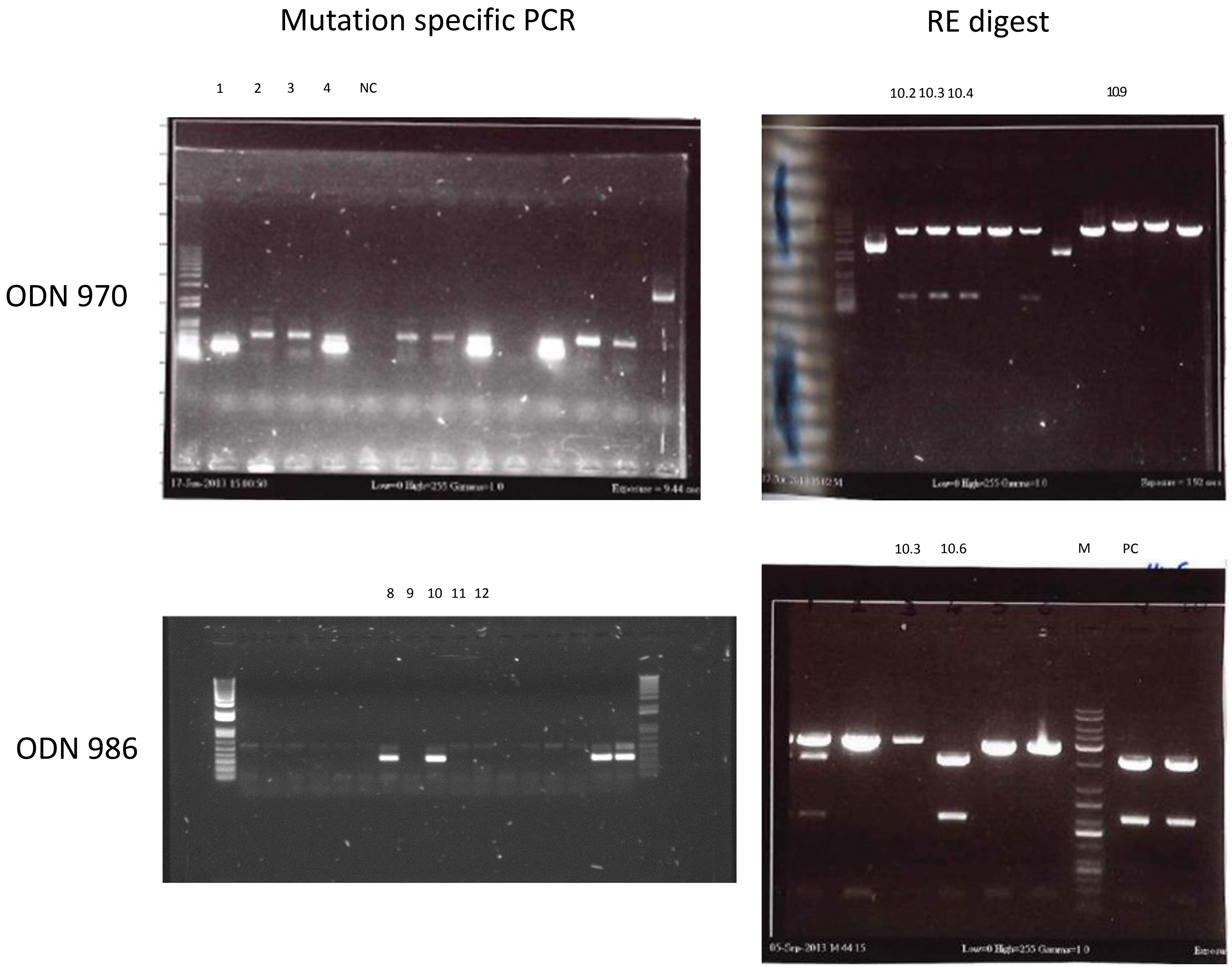


**Supplementary Fig. S7: Uncropped gel images of Fig. 3**

**Supplementary Table S2:** Summary of deep sequencing results of ZFN/ODN 986 and TALENs/ODN 986 injected embryos

| Sample | Material | Time | % Edited allele[a] | % WT Allele[a] | Total Joined[b] |
|---|---|---|---|---|---|
| ZFNs | | | | | |
| Z1 | DNA | 8 h | 15 | 47 | 56,315 |
| Z2 | DNA | 8 h | 23 | 73 | 49,772 |
| Z5 | DNA | 8 h | 2 | 73 | 70,994 |
| Z6 | DNA | 8 h | 63 | 35 | 42,121 |
| Z3 | DNA | 18 h | 24 | 69 | 86,132 |
| Z4 | DNA | 18 h | 0.2 | 71 | 86,419 |
| Z7 | DNA | 18 h | 0.3 | 91 | 78,211 |
| Z8 | DNA | 18 h | 17 | 75 | 22,238 |
| Z25 | RNA | 8 h | 0.1 | 91 | 160,600 |
| Z26 | RNA | 8 h | 0.1 | 88 | 79,196 |
| Z21 | RNA | 18 h | 0.3 | 93 | 29,605 |
| Z23 | RNA | 18 h | 0.6 | 89 | 170,964 |
| TALENs | | | | | |
| T1 | DNA | 18 h | 100 | 0 | 203,280 |
| T2 | DNA | 18 h | 96 | 4 | 10,976 |
| T3 | DNA | 18 h | 21 | 53 | 148,289 |
| T4 | DNA | 18 h | 3 | 82 | 43,826 |
| T5 | DNA | 18 h | 99 | 1 | 178,297 |
| T6 | DNA | 18 h | 88 | 10 | 111,171 |
| T7 | DNA | 18 h | 11 | 62 | 5,520 |
| T8 | DNA | 18 h | 12 | 73 | 145,084 |
| T9 | DNA | 18 h | 13 | 68 | 216,754 |
| T10 | DNA | 18 h | 15 | 75 | 248,492 |
| T11 | DNA | 18 h | 71 | 11 | 120,139 |
| T12 | DNA | 18h | 78 | 0 | 88,138 |
| T13 | DNA | 18 h | 38 | 57 | 179,048 |

| | | | | | |
|---|---|---|---|---|---|
| T14 | DNA | 18 h | 100 | 0 | 63,426 |
| T15 | DNA | 18 h | 22 | 64 | 145,874 |
| T16 | RNA | 18 h | 100 | 0 | 109,316 |
| T17 | RNA | 18 h | 3 | 83 | 33,089 |
| T18 | RNA | 18 h | 31 | 56 | 20,077 |
| T19 | RNA | 18 h | 15 | 58 | 75,459 |

[a] percentage of total number of joined reads

[b] total number of joined paired end sequence reads

**Supplementary Table S3:** Primer sequences

| Primer | Sequence[a] | Application |
|---|---|---|
| 840 | AGGCCTCCTATTGTCCTCGT | Amplification of LGB locus |
| 841 | GCAAAGGACACAGGGAGAAG | Amplification of LGB locus |
| 842 | ATGAAGTGCCTCCTGCTTG | TaqMan PCR primer |
| 843 | CACCCTCGAACCTTCTGGAT | TaqMan PCR primer |
| 871 | TCATYGTCACCCAGACCA | TaqMan probe |
| 970 | CTTGCCCTGGCCCTCACYTGTGGCGCCCAGGCCCTCATYGTCACCCTCTAGAG ACCATGAAGGGCCTGGATATCCAGAAGGTTCGAGGG | Homology repair template |
| 986 | GCAGCCATGAAGTGCCTCCTGCTTGCCCTGGCCCTCACYTAGGCCCTCATYGT CACCCAGACCATGAAGGGCCTGGATATCCAGAAGGTTCGAGGGTGSC | Homology repair template |
| 987 | CCCTCATYGTCACCCTCT | Primer specific for ODN 970 mutation |
| 994 | CTGGCCCTCACYTAGGCCCT | Primer specific for ODN 986 mutation |

[a] The nucleotide Y specifies a pyrimidine base (C or T) and the nucleotide S stands for either C or G according to the polymorphic sequences present in the main two *LGB* variants A and B
